# The magnitude and factors associated with work-related back and lower extremity musculoskeletal disorders among barbers in Gondar town, northwest Ethiopia, 2017: A cross-sectional study

**DOI:** 10.1371/journal.pone.0220035

**Published:** 2019-07-22

**Authors:** Tesfaye Hambisa Mekonnen

**Affiliations:** Department of Environmental and Occupational Health and Safety, Institute of Public Health, College of Medicine and Health Sciences, University of Gondar, Gondar, Ethiopia; University of Arkansas for Medical Sciences, UNITED STATES

## Abstract

**Background:**

Work-related back and lower extremity disorders often present remarkable health and economic burdens on societies. Occupational barbers are usually neglected in research and policy actions, mainly in developing countries, and are hence more vulnerable to the conditions. So far, information about the factors influencing back and lower extremity disorders among barbers in Ethiopia is unknown. Therefore, the aim of this study was to determine the prevalence and factors affecting back and lower extremity disorders among barbers in Gondar town, Ethiopia.

**Methods:**

A cross-sectional study was conducted from April to May 2017. A sample of 434 barbers recruited using the systematic random sampling technique. A pre-tested standardized Nordic Musculoskeletal questionnaire was interviewer-administered for data collection. Data were analyzed using statistical package for social sciences (SPSS) version 20. The significance of associations was evaluated at ≤0.05 p-value with a 95% confidence intervals (CI) and adjusted odds ratios (AOR).

**Results:**

The response rate was 98.8% (N = 429). The mean age and mean length of employment were 26.38 (standard deviations (SD) ± 4.78) and 4.91 years, respectively. The prevalence of work-related low back pain in the previous 12 months and in the last 7 days was 55.7% (N = 239) [95% CI (51.0, 60.4)] and 32.6% (N = 140), respectively. About 40.6% (n = 97) of the participants with back pains indicated their activities were limited. The prevalence of knee/leg and ankle pain was 39.4% (N = 169) and 25.6% (N = 110), respectively. Out of the participants, 17% (n = 41) sought treatment services. Less than half, 40.6% (n = 97) said they perceived high disability, while 38.1% (n = 91) explained their pain was intense (severe). Age [AOR: 2.001; 95% CI (1.174, 4.346)], alcohol use [AOR: 2.283; 95% CI (1.376, 3.789)], lack of safety training [AOR: 0.110; 95% CI (0.032, 0.271], working posture [AOR: 0.142; 95% CI (0.045, 0.215)], and length of employment [AOR: 1.650.132; 95% CI (1.107, 2.140] were significantly associated factors.

**Conclusions:**

Back and lower extremity musculoskeletal pain and disability were found to be prevalent among Ethiopian barbers and to be associated with age, alcohol use, safety training, work postures, and length of employment. We believe that programs for management of musculoskeletal disorders need to address these factors.

## Background

The barbershop sector is one of the precarious occupations, which is inherently associated with several workplace risk factors [1]. The combination of exposures to various physical, chemical, ergonomic, psychosocial, and biological hazards in this occupation is usually noticeable [2–4]. Consequently, barbers are often very susceptible to the various adverse health outcomes, like work-related musculoskeletal disorders [5]. In this sector, work characteristics, such as fixed or constrained body positions, continual repetition of movements, force concentration on small parts of the body, like the hand or wrist, pace of work that does not allow adequate recovery between movements, vibration, and temperature lead to the developments of musculoskeletal disorders [6–9].

Work-related musculoskeletal disorder (WMSD) is one of the major public health concerns resulting from the growing demands of healthcare service utilization, temporary and permanent disability, and reduced quality of life it usually incurs [8,10]. Moreover, it is a contemporary occupational health problem, representing reduced productivity, absence from work, and rising compensation premiums [10,11]. For instance, in the United Kingdom (UK), an estimated 6.6 million working days were lost due to work-related musculoskeletal disorders, constituting 24% of all days lost due to work-related ill-health in 2017/18 [12]. Of these, work-related back and lower limb disorders accounted for 2.2 and 1.7 million days lost, respectively. A study in Turkey also demonstrated that WMSD account for 34% of all work days lost due to occupation-related diseases [8].

Barbershop occupations are also associated with the hazardous nature of working conditions, exposing barbers to the risks of musculoskeletal disorders [2,12,13]. Barbers often stand long hours and bend/twist their backs forward or sideways during their activities that boosts the developments of back and lower limb disorders [14,15]. Thus, scholars conclude that the prevalence of work-related back and lower extremity disorders among barbers is pervasive. For instance, recent studies showed that prevalence was 76.3% in Nigeria [16], 27.4% in Turkey [5], and 39% in Brazil [17]. Similarly, a study conducted in Greece reported a 28% prevalence of knee pain [18]. Studies in Iran showed that the prevalence of leg pain was 31% [15] and 66.5% [19]. Other studies reported the prevalence of lower extremity disorders to be between 52.3 and 67.7% [20,21].

Several investigations reveal that a number of occupational factors determine the developments of back and lower limb disorders. In fact, literature demonstrates that socio-demographic factors, like sex, age, marital status, and experience [8,22,23] markedly influence the experience of back and lower limb musculoskeletal pains. Moreover, workplace factors, including working hours, job tenure, type of activity (static and/or dynamic), shift work, safety training, working posture, rest breaks [19,23–28], and psychosocial factors, such as job satisfaction and stress [22,24,26,29] predict the likelihood of sustaining back and lower extremity musculoskeletal disorders. Behavioral styles, like alcohol consumption [30], physical activities [24,29,31] and body mass index [19,27,32], and previous history of systemic illnesses [22,33] were also reported as potential risk factors of back and lower limb disorders.

In Ethiopia, informal sectors, including barbershop industries, are vastly growing. Health and safety protection of the workers in these sectors is, however, usually disregarded both in research and policy action. In these sectors, despite the ever mounting manpower in poor working conditions that predispose them to various disorders, research about prevalence and factors associated with work-related back and lower extremities musculoskeletal disorders is scant. Therefore, the objective of this study was to explore the prevalence and work-related factors of back and lower extremity musculoskeletal disorders among barbers in Gondar town, northwest Ethiopia.

## Methods

### Study design and period

A cross-sectional study was conducted from April to May 2017 to explore the prevalence and occupational factors associated with back and lower extremities musculoskeletal disorders among barbers in Gondar town.

### Study setting and area

This study was conducted on barbers in Gondar town, northwest Ethiopia. The town is one of the tourist destinations in the Amhara Regional State, northwest Ethiopia, 747 km from Addis Ababa, the capital of the country. According to the 2007 Central Statistical Agency (CSA) report of Ethiopia, the town had a total population of 207,044 of whom 108,924 were women. During the data collection, there were 1150 barbershop professionals in the about 20 kebeles.

### Source and study population

All barbers in Gondar town were the source population. The randomly selected barbers in the selected kebeles were our study population. Barbershop professionals who had worked for at least 12 months in the study area prior to the study were included, whereas those who were on sick, annual, maternity, and family leaves and those who had previous history of back pains, car accidents, and injuries were excluded.

### Sample size determination

Sample size was calculated using OpenEpi software with population (N) of 1150 barbers, 50% (P) hypothesized frequency of outcome variable, and 4% absolute precision with 95% CI at Z = 1.96 critical value using the formula: *n* = [Np (1-p)]/ [(d^2^/Z^2^_1-α/2_*(N-1) + p*(1-p)]. Assuming a 10% for no responses, the final sample was = 395+39 = 434.

### Data collection tools and procedures

The systematic random sampling technique was used to recruit eligible samples. An interviewer-administered questionnaire was employed for data collection. We assessed self-reported (Yes/No) prevalence of back and lower extremity musculoskeletal disorders using a standardized Nordic Musculoskeletal Questionnaire. The instrument was previously endorsed as appropriate for interview data collection technique [34]. The standardized questionnaire has been applied in intensive studies in the literature, including Ethiopia [33,35,36]. The satisfaction of barbers with their jobs was evaluated using a generic job satisfaction scale questionnaire [37]. We also assessed perceived job stress using the new job stress scale [38]. Perceived severity and disability of conditions were evaluated according to the Von Korff pain severity grading [39]. The other detailed contents of the questionnaire comprised of four parts. The first part contained socio-demographic factors, like sex, age, religion, educational status, marital status, monthly salary, and work experience. The second category covered organizational/work-place factors, including working hours per day, health and safety training, number of customers per day, pre and periodic medical examinations, shift work, working posture, and rest break. The third component encompassed health and psychosocial factors, like previous history of systemic illnesses, job satisfaction, and job stress. The behavioral style part covered details of factors, like physical exercise (Yes/No), smoking (Yes/No), handedness (right/left), and body mass index (BMI) (Weight divided by height square).

### Data quality control

The designing of appropriate data collection tools was given priority. The questionnaire was first developed in English and translated in to local language ‘Amharic’ and back to English by language experts to ensure consistency. Eight environmental and occupational health and safety final year students in the College of Medicine and Health Sciences at the University of Gondar were involved in data collection after they took adequate training and orientation. Four well experienced supervisors were recruited from the Environmental and Occupational Health and Safety department. The data collectors and supervisors took the orientation on issues relating to the clarity of the questions, objectives of the study, confidentiality of information, and the voluntary involvement (consent) in the study. The principal investigator supervised both data collectors and supervisors. To test the validity and reliability of the questionnaire, a pre-test was conducted on 18 samples in a kebele not included in the final survey. A few modifications, such as minimizing the number of questions were made, and some misinterpretations and ambiguities corrected, based on the pretest analysis.

### Methods of data analysis

The data were manually cleaned for completeness, coded, and entered in to EPI info version 7.1.5.2 and exported to SPSS version 20 software for analysis. Frequency distributions, percentages, means, and standard deviations were used to describe results. The reliability of the standardized Nordic Musculoskeletal Questionnaire was tested using Cronbach’s Alpha and found a reliable Cronbach’s Alpha = 0.79. The 10- items generic job satisfaction scale questionnaire was also examined for reliability and Cronbach’s Alpha was found to be 0.911. The 22 item job stress scale questionnaire was also checked for reliability and found Cronbach’s Alpha = 0.87. The instruments were, therefore, tolerable for their consistencies in repeating what have previously been measured using the tools. The associations between the dependent variable (back and lower extremities musculoskeletal disorders) and independent variables were examined using a binary logistic regression analysis. Accordingly, explanatory variables with a < 0.2 p-value in a bivariate analysis were exported to a multivariable logistic regression model to further investigate the potential effects of confounders. A forward variable selection method was used to drag variables in to the multivariable logistic regression model. The goodness of fit model was checked by Hosmer and Lemeshow and found to be good model fitness with a 0.818 p-value. The odds ratios with 95% confidence intervals (CI) were calculated to evaluate the strength and a cut off ≤ 0.05 p-value was established to ascertain the significance of associations.

### Operational definition

**Back and lower extremity musculoskeletal disorders:** Having had trouble (ache, pain and discomfort) in low back (small of the back), one or both hips/thighs, one or both knees, one or both ankles/feet any time during the last 12 months [34]

**Perceived severity:** A pain intensity score of ≥50 or < 3 disability points [39]

**Perceived disability:** A pain disability point score of 3–6 points [39]

**Stressed worker:** The new job stress scale score above the overall mean score [38]

**Job satisfied worker:** The generic job satisfaction scale score of 32 or above [37]

### Ethics approval and consent to participate

Ethical clearance was obtained from the Institutional Ethical Review Board (IERB) of the University of Gondar, College of Medicine and Health sciences, Institute of Public Health (Reference #: EOHS/435/2009). We communicated the letter to each owners of the barbershops selected for inclusion. We also obtained verbal informed consent from each respondent. Confidentiality of data was maintained. Only aggregate data were used. Any involvement in the study was carried out with the full consent of the person willingly participating in the study.

## Results

### Socio-demographic characteristics

A total of 429 barbers participated with a response rate of 98.8%. The majority, 86.9% (N = 373) of the participants were males. A high proportion, 85.8% (N = 368) of the respondents’ age was ≤ 30 ranging from 17 to 50 with a mean of 26.38 (SD ±4.78) years. About 10.5% (N = 45) of the participants said they could not read and write, whereas 49.7% (N = 213) attended secondary schools. More than half, 60.6 (N = 260) of the participants were unmarried. Few, 7% (N = 30) of the interviewees’ monthly salary was < ETB 1100 and 57.6% (N = 247) reported their monthly salary was ETB 1101–1700 **(Table 1)**.

**Table 1:** Socio-demographic characteristics in relation to back and LEDs, Ethiopia, 2017.

Variables (N = 429)	Frequency	Percentage (%)	P-value
**Sex**			
Male	373	86.9	
Female	56	13.1	0.177
**Age**			
≤ 30 years	368	85.8	
>30 years	61	14.2	0.001
**Marital status**			
Married	134	32.2	
Single	260	60.6	
Divorced	35	8.2	0.060
**Educational status**			
Cannot read and write	45	10.5	0.178
Primary education (1–8)	72	16.8	
Secondary education (9–12)	213	49.7	
Above secondary education	99	23.1	
**Monthly salary**			
<1100 ETB	30	7.0	0.149
1101–1700 ETB	247	57.6	
>1700 ETB	152	35.4	
**Religion**			
Orthodocs	348	81.1	0.125
Catholic	12	2.8	
Protestant	34	7.9	
Muslim	35	8.2	
**Experience**			
≤ 5 years	308	71.8	
>5 years	121	28.2	0.008

**Keys:** ETB = Ethiopian birr (currency); N = number; LEDs = lower extremity disorders

### Behavioral and psychosocial characteristics of the participants

Among the respondents, 35.9% (N = 154) reported they were alcohol users. A total of 42.0% (N = 180) participants reported they were chat chewers, whereas 81.1% (N = 348), 13% (N = 567), and 5.6% (N = 24) were never smokers, current smokers, and passive smokers, respectively. Regarding physical exercises, 30.5% (N = 130) reported they performed physical exercise. About 10.0% (N = 43) stated they had exercises for 1–2 hours per day, 9.6% (N = 41) for >2 hours per day, 7.2% (N = 31) 1–3 times per week, and 4.2% (N = 18) every day. Thirty-one percent (N = 137) of the barbers pointed out they had systemic illnesses. Out of the interviewees, 66.7% (N = 286) explained they were not satisfied with their jobs, whereas 24% (N = 103) reported they perceived stress due to their work.

### Occupational characteristics of the study participants

About more than half, 60.4% (N = 259; p = 0.258) of the participants showed that their pattern of employment was temporary while the remaining reported they were permanent. The majority of barbers, 79% (N = 339; p = 0.077) said they were employed by others and the remaining indicated they were self-employed. Seventy-four percent (N = 319; p = 0.0001) of the participants indicated they worked > 8 hours per day. Only 7.9% (N = 34; p = 0.859) of the barbers reported that they took safety training, and 22.1% (N = 95), 25.2% (N = 108), and 52.7% (N = 226; p-value = 0.012) reported their payment scheme was monthly, hourly, and per piece, respectively. Forty-four percent (N = 215; p = 0.031) revealed that they did not use rest break at their workplaces. The respondents presented that 7.5% (N = 32), 25.6% (N = 110), and 66.9% (N = 287; p = 0.256) of them spent 1–5, 6–10, and > 10 hours standing per day, respectively. The study also found that 37.1% (N = 159; p = 0.016) of the participants indicated their working posture was awkward/bending/twisting/, 41.7% (N = 179) static posture/frequent standing, and 21.2% (N = 91) alternate/flexible postures.

### Prevalence of back and lower extremity musculoskeletal disorders

The prevalence of low back pain in the past 12 months and 7 days was 55.7% (N = 239) [95% CI (51.0, 60.4)] and 32.6% (N = 140), respectively. There was no statistically significant difference between male and female participants (p = 0.603). About 42.2% (n = 101; p = 0.001) of the respondents with back and lower extremity disorders indicated that they experienced the symptoms in more than a single body site (co-morbid). Of the participants, 40.6% (n = 97; p = 0.0001) indicated their activities were limited because of the complaints. Lower body sites represented with the symptoms included hip/thigh 28.9% (N = 134), knee/leg 39.4% (N = 169), and ankle/feet pains 25.6% (N = 110). About 45.6% (n = 109) of the participants with lower extremity disorders demonstrated that they experienced them in the last 7 days and 21.3% (n = 51) were prevented from their activities due to the conditions. Out of the total indicated disorders of back and lower body sites, 21.3% (n = 51) revealed they sought treatment services and 40.6% (n = 97) said they perceived high disability, while 38.1% (n = 91) explained their pain was intense (severe).

### Factors associated with back and lower extremity musculoskeletal disorders

A bivariate analysis showed that age, working hours, work experience/length of employment, lack of health and safety training, alcohol drinking, working posture, educational level, job satisfaction, history of systemic illness, and rest break were the factors substantially associated with work-related back and lower body disorders.

After controlling for confounders in a multivariable logistic regression analysis, age, length of employment, alcohol use, lack of safety training, and working posture remained to considerably influence the developments of back and lower extremity disorders. Accordingly, participants aged > 30 years were 2.001 times more at risk for developing back and lower extremity disorders than those aged ≤ 30 years [AOR: 2.001; 95% CI (1.174, 4.346)]. The odds of experiencing back and lower extremity disorders were 2.283 times more likely among alcohol users than non-users [AOR: 2.283; 95% CI (1.376, 3.789)]. Moreover, 89% of the likelihood of developing back and lower extremity disorders was prevented among participants who took safety and health training than those who did not [AOR:0.110; 95% CI (0.032, 0.271)]. Barbers who had worked in flexible/alternative work postures were 85.8% less likely to develop back and lower extremity disorders than those who worked in static/frequent standing work postures [AOR: 0.142; 95% CI (0.045, 0.215)]. Back and lower extremity disorders were 1.650 times more likely to be experienced among barbers with >5 years of employment compared to those with ≤ 5 years **(Table 2).**

**Table 2:** Factors associated with back and lower extremity disorders among barbers, 2018.

Variables (N = 429)	Back and lower extremity disorders				
	Yes	No	Crude OR (95%CI)	Adjusted OR (95%CI)	P-value
**Age**					
≤ 30 years	196	172		1	
>30 years	43	18	2.096(1.482, 4.925)	2.001 (1.174,4.346)	0.0001**
**Level of education**					
Cannot read and write	55	31	1.533(0.970,2.535)	1.025(0.157, 3.312)	0.103*
Can read and write	184	159	1	1	1
**Work experience**					
≤ 5 years	159	149	1		
>5 years	80	41	1.828(1.417,3.433)	1.650 (1.107, 1.140)	0.001*
**Working hours/day**					
≤ 8 hours	50	59	1	1	
>8 hours	189	131	1.702(1.087,2.627)	1.345(0.813, 2.226)	0.071*
**Safety training**					
No	209	186	0.498(0.183,1.243)	0.110(0.032, 0.271)	0.0001*
yes	30	4	1	1	
**Alcohol drinking**					
No	132	143	1	1	
Yes	107	47	2.466(2.435, 5.973)	2.283 (1.376, 3.789)	0.000**
**History of systemic illness**					
No	156	136	1	1	
Yes	83	54	1.339(1.344, 3.149)	1.028(0.161, 3.901)	0.182*
**Working posture**					
Bending and twisting/awkward posture	84	75	0.803(0.543, 1.649)	0.126(0.134, 1.134)	0.102*
Static work/frequent standing	102	77	0.949(0.543, 1.649)	0.142 (0.045, 0.215)	0.0001**
Alternative postures	53	38	1	1	
**Rest break**					
No	111	104	0.717(0.505, 1.100)	1.035(0.613, 1.747)	0.122*
Yes	128	86	1	1	
**Job satisfaction**					
Satisfied	86	57	1	1	
Not satisfied	153	133	0.762(0.977, 2.244)	1.229(0.702, 2.104)	0.140*

Keys:-

* = Significant at a <0.2 p-value in a bivariate analysis

** = Significant in multivariate analysis at a <0.05 p-value

ETB = Ethiopian birr; N = Number; OR = Odd ratio; CI = Confidence interval; N = number; LEDs = lower extremity disorders

## Discussion

This study employed a workplace-based cross-sectional design to evaluate the prevalence and factors associated with back and lower limb musculoskeletal disorders among barbers in Gondar town, northwest Ethiopia. The prevalence of low back pain in the previous 12 months was 55.7% (N = 239) [95% CI (51.0, 60.4)]. This finding was relatively equivalent to that of a study conducted in Greece (53%) [18]. The possible reason for this might be the similarity of the working environment and conditions of barbershop occupations across countries. Another probable suggestion might be the fact that informal sectors are often not included in the national labor laws and regulations in many countries. The exclusion of these sectors in the national laws probably leads to poor access to occupational health and safety services, which further proliferates the situations. However, our finding indicates a higher prevalence compared to the studies in the UK (4.9%) [40] and Brazil (39%) [17]. On the other hand, we found a lower magnitude of low back pain disorders compared to a study in Nigeria (76.3%) [16]. The difference could be due to the availability/unavailability of workplace health and safety services, illness and injury management and reporting procedures, and data collection methods.

Our result shows that the level of ankle/feet pain was 25.6% (N = 110) [95 CI (21.4, 29.6)] and that of knee/leg 39.4% (N = 169) [95 CI (35.0, 44.3)]. A previous report in Iran (70.7%) found a higher prevalence of ankle/feet pain than our investigation did [19]. The study also reported a higher magnitude (66.5%) of knees/legs pain. About 28.9% (N = 124) [95 CI (24.7, 33.3)] of the participants in our study indicated they had experienced pain in their hip/thigh body sites. This result was higher than the report in Nigeria (16.6%) [16]. The discrepancies could be due to differences in data collection methods, injury and illness management, and reporting procedures.

The result of the multivariable regression analysis demonstrated that age markedly contributed to the development of back and lower extremity disorders. This result was in agreement with the results of other studies [16,19,41]. The probable explanation for these similarities is the fact that the biological/functional structures of the human body, particularly those related to supportive structures, like muscles, joints, nerves, ligaments, and tendons would tend to degenerate as age increases. This could be likely to induce the reduced structural functional capacities of the workers. Similar explanations have been provided by previous investigations [42,43]. The other possible reason could be the effect of aging or a cumulative effect of workload on the musculoskeletal systems through years of employment services. Improvements in modern human lifestyles would also be likely to increase workers’ chance of extended stay in the employment world, resulting in the current domination of working workplaces for aging workers.

The current study identified that the length of employment/work experience is an important determinant of back pains and lower extremity disorders. This result was supported by other scholastic works [16–18,41]. The possible explanation might be the fact that employees with comparatively longer duration of employment are often victims of the effects of cumulative exposure to ergonomic and other workplace hazards. Another possible reason might be that workers with longer duration of employment might neglect the possible protection mechanisms of potential health and safety risk factors at their workplace, relying on the length of employment/workplace adaptation as a means of protection from adverse effects.

Alcohol consumption was found to be the other significant factor of back and lower extremity complaints. This result corroborates previous studies [16,17,30]. The plausible reason may be that alcohol drinking is one of the common health risk behaviors that might deteriorate the normal functional capacities and defense mechanisms of the body. A more possible explanation is that alcohol drinking might negatively influence the behavior of people, often prohibiting from exercising a healthy life-style, such as physical exercise.

The absence of health and safety training was the other significant factor for back and lower extremity conditions. The proximal association between safety training and LBP has rarely been studied. Workers’ health and safety awareness and training could however, play a great role in hazard and risk prevention and control measures. A study in Ethiopia was concurred with this result [19]. Safety training is more likely to promote workplace cultures of safety. Training targeting healthy life-styles might also result in behavioral changes of employees leading them to enjoy physical exercises, which could in turn lead to a reduced probability of back pain disorders.

The multivariate analysis also revealed that working posture considerably contributed to the risk of developing back and lower extremity pains. This finding was consistent with those of other studies [16–18,28,43,44]. It could be explained that awkward postures, such as twisting and bending and static postures, like prolonged standing, might impose stress on specific body parts by exerting pressure on locomotive body structures, leading to physical and functional impairments. A static/inflexible nature of working position for an extended time might also enhance muscle stiffness.

The distinguishing nature of the current study is that it pioneered the exploration of the prevalence and work-related factors leading to back and lower extremity disorders that usually experienced by people engaged in the cutting and shaving industry in Ethiopia. The potential occupational-related factors contributing to the conditions were replicated and the government and other stakeholders could benefit from it for policy design and implementations. However, the self-report data collection method employed in this study might be a limitation, as recall bias and under reporting could be anticipated. The temporal relationships between work-related symptoms of back and lower extremity disorders and the hairdressing-job related factors should be treated with caution as the study used a cross-sectional design. The lack of job posture analysis that could help ascertain the degree to which awkward postures/bending and twisting /are determined is other limitation of this study. Moreover, it might be difficult to generalize the findings, because the study dealt with only a specific segment of workforce. Therefore, future investigations with larger samples and multiple sectors with strong designs, such as longitudinal studies, are greatly suggested.

## Conclusions

Back and lower extremity musculoskeletal pain and disability were found to be prevalent among Ethiopian barbers and to be associated with age, alcohol use, safety training, work postures, and length of employment. We believe that programs for management of musculoskeletal disorders need to address these factors.

## Supporting information

S1 File This is data set used in analysis. (XLSX)

## Abbreviations

AOR: adjusted odds ratio
BMI: Body mass index
BSc: Bachelor of Science
CI: Confidence interval
ETB: Ethiopian Birr
Km: Kilometers
MPH: Masters of public Health
MSc: Masters of Science
OR: Odds ratio
SD: standard deviation
UK: United Kingdom
US: United States of America
LEDS: lower extremity disorders
